# Combined Boron Toxicity and Salinity Stress—An Insight into Its Interaction in Plants

**DOI:** 10.3390/plants8100364

**Published:** 2019-09-23

**Authors:** Anamika Pandey, Mohd Kamran Khan, Erdogan Esref Hakki, Sait Gezgin, Mehmet Hamurcu

**Affiliations:** Department of Soil Science and Plant Nutrition, Faculty of Agriculture, Selcuk University, Konya 42079, Turkey; anamika.biotech@gmail.com (A.P.); sgezgin@selcuk.edu.tr (S.G.); mhamurcu@selcuk.edu.tr (M.H.)

**Keywords:** combined stress, boron, novel stress, salinity, shared response

## Abstract

The continuously changing environment has intensified the occurrence of abiotic stress conditions. Individually, boron (B) toxicity and salinity stress are well recognized as severe stress conditions for plants. However, their coexistence in arid and semi-arid agricultural regions has shown ambiguous effects on plant growth and development. Few studies have reported that combined boron toxicity and high salinity stress have more damaging effects on plant growth than individual B and salt stress, while other studies have highlighted less damaging effects of the combined stress. Hence, it is interesting to understand the positive interaction of this combined stress so that it can be effectively employed for the improvement of crops that generally show the negative effects of this combined stress. In this review, we discussed the possible processes that occur in plants in response to this combined stress condition. We highly suggest that the combined B and salinity stress condition should be considered as a novel stress condition by researchers; hence, we recommend the name “**BorSal**” for this combined boron toxicity and high salinity state in the soil. Membrane-bound activities, mobility of ions, water transport, pH changes, transpiration, photosynthesis, antioxidant activities, and different molecular transporters are involved in the effects of BorSal interaction in plants. The discussed mechanisms indicate that the BorSal stress state should be studied in light of the involved physiological and molecular processes that occur after B and salt interaction in plants.

## 1. Introduction

In modern research, the majority of abiotic stress-based responses that are verified in laboratories or controlled conditions occur differently under field conditions [1]. One of the factual reasons underlying this phenomenon is the simultaneous existence of several abiotic stresses in the field that altogether act as a novel stress factor and may act differently in plants [2,3]. Hence, efforts should be made to mimic these combined stress conditions in laboratory experiments and understand the physiological and the molecular mechanisms underlying them.

Boron (B) and salinity are two drastic individual abiotic stress conditions largely responsible for crop losses [4,5]. B toxicity is one of the major challenges that restrict crop production worldwide [6,7,8,9,10]. As reported by Nable, Bañuelos, and Paull [8], vast regions of Lake California, Israel, India, northern Chile, Peru, Malaysia, the Middle East, and South Australia showed high soil B content [6,9,11,12,13,14,15,16].

High soil salinity affects 20–50% of irrigated agricultural land with an approximate annual economic drop of 12.6 billion USD [17]. However, these stress conditions not only occur individually but also co-exist in alkaline soils in low rainfall regions [18,19,20,21,22,23]. Although there are several reports on crop yield reduction under this combined stress condition, other studies have reported its less damaging effect on the growth and the development of crops; thus, a positive interaction between the two types of stresses can be considered when simultaneously present.

In the last few years, attempts have been made to understand the combined effects of these stresses on different crops, but no clear conclusions have been obtained regarding their interactive effects [21,24,25,26]. Hence, in this review, we discuss the potential mechanisms involved in this combined stress condition of high B and high salt (**BorSal**) so that agricultural solutions for this issue can be pursued by considering the involved mechanisms. To develop a genetic engineering-based amendment, it is extremely crucial to understand the involved mechanisms at the molecular level.

## 2. Combined Stress on Plants—A Major Problem of Today’s World

Because of the continuously changing environment, plants are being increasingly exposed to combinations of different stresses as compared to individual stresses. The simultaneous occurrence of abiotic stresses instead of individual stress condition can be more fatal to crops [27,28,29]; however, this is not always applicable.

Plants show both unique and shared responses to adapt themselves to combined stress conditions [30]. On the one hand, combined stresses negatively affect plant growth; on the other hand, they sometimes facilitate the adaptation strategy of plants and protect them [27,31,32,33,34]. Thus, combined stress condition should be considered as a novel stress and should be investigated in detail [35]. It is necessary to understand the interaction between any two simultaneously occurring stresses and responses of plants to such stresses. If the combined stress is less damaging for plants than the individual stress condition, then we could consider it as a “positive effect”. If the combined stress has a more damaging effect, then we could consider it as a “negative effect”. Several tolerance mechanisms have already been proposed for protecting crops against simultaneous environmental stresses [36,37] with the main emphasis on drought and heat stress as a combined stress condition [38,39,40,41]. However, studies addressing the combined B and salt stress condition are limited. 

## 3. Prevalence of BorSal as a Combined Stress Condition

B stress affecting crop productivity is often accompanied by soil salinity. Crops in arid or semiarid agricultural areas facing high salinity problems often experience B stress due to limited leaching [20,26,42,43,44,45,46,47,48,49,50]. Because of its soluble nature, B accumulates as sodium salts, especially in regions with poor drainage [51]. BorSal co-occurs in plants mostly due to irrigation with water containing high levels of B and salts [8] or through the growth of plants in naturally B and salt-rich soil [8,52].

Although some studies have been conducted to determine the effect of B and salinity as individual stresses [44,53,54,55,56,57,58,59], research focusing on the simultaneous interactive effects of both the stresses is limited [50,60].

Few reports have shown negligible effects of combined BorSal stress on the shoot weight of different cultivars [54,61,62,63], while other studies documented reduced tolerance to B in the presence of salinity stress [24,45,53,60,64,65].

Increasing salt concentrations may add to the negative (damaging) effect of B toxicity on plant growth. In wheat, high B and salt concentrations can be more disastrous to plants in the form of combined stress having a suppressive effect on shoot dry weight and grain yield [43]. However, this negative effect is not always dependent on the shoot B concentration in plants [43,45]. Few studies have demonstrated an increase in shoot B concentration with the increase in salinity [45], while other studies have demonstrated a decrease in B accumulation in both shoots and grains under high salt concentrations [43,45]. Bingham et al. [54] reported statistically significant effects of individual B and salinity stresses on growth parameters and leaf B concentrations of spring wheat, while B–salinity interaction did not have considerable effects on the plants. These variations can be attributed to several factors such as high Na or Ca ions in the source of salinity or the soil pH that largely controls the B uptake and the shoot B concentrations of plants. Moreover, the source of salinity (NaCl or Na_2_SO_4_) also causes variations in B accumulation in plants. A number of studies have investigated the interactive effects of NaCl and Na_2_SO_4_ salinity under B toxicity and have obtained varying results [53,66,67,68,69,70,71]. Generally, NaCl salinity was more disastrous for plants than Na_2_SO_4_ salinity [66,72].

**Figure 1:**
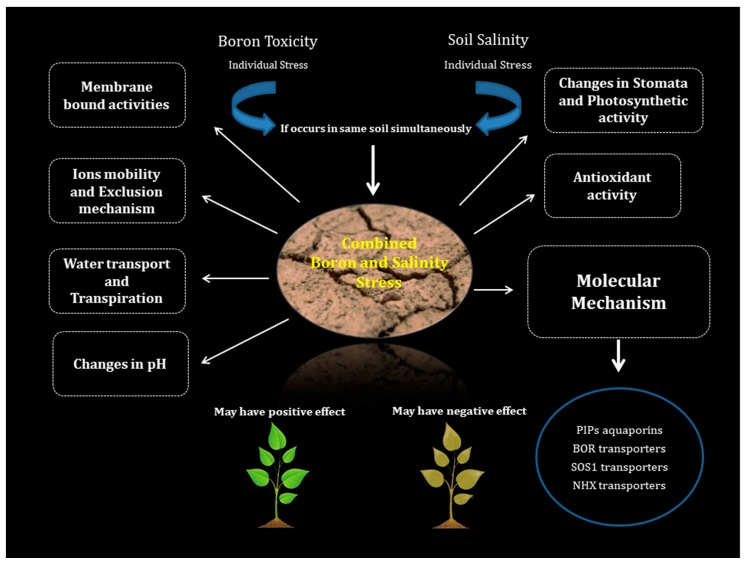
Different mechanisms involved in increasing the tolerance or the susceptibility of plants towards combined B toxicity and salinity stress condition.

Plants acclimatize to this BorSal condition with the help of both “unique” and “shared” stress responses. Few responses are unique to B and salinity stress, while others are common to both the stresses. B absorption, exclusion mechanism, membrane bound activities, and chemical changes in plants are among the important channels involved in reducing B toxicity under saline conditions [53,60,70,73,74] (Figure 1). Moreover, there are several other factors affecting B accumulation in plants that include salt tolerance capacity of a particular genotype, type of species grown, and growth environment [53].

The nature of the interaction of salinity and B in plants is not conclusive and needs to be thoroughly understood [75]. Recently, in 2015, a book was published that describes the alterations in plant growth stages to cope with the effect of several stress combinations [28]. Although different stress combination types are discussed in that book, BorSal stress remains unexplored. Amid diverse stress combinations that arise in field environments, the interaction between B and salinity stress is one of the least studied mechanisms. Hence, in the present article, we discuss the morpho-physiological and the molecular responses of plants towards this stress combination and the potential mechanism underlying such responses.

## 4. BorSal Interaction in Plants in Terms of Membrane Bound Activities

**Figure 2:**
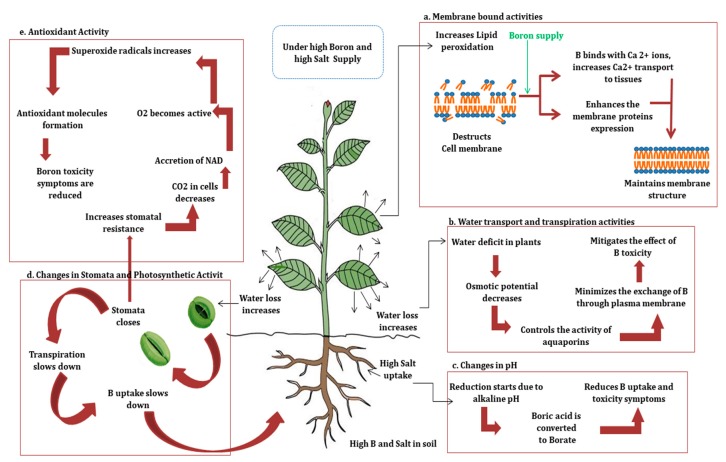
An insight into the mitigating effect of combined high B and salt stress on the toxicity symptoms in plants. Toxicity symptoms are reduced via different pathways/strategies involved. (**a**) Membrane bound activities, (**b**) water transport and transpiration activity, (**c**) changes in pH, (**d**) changes in stomatal and photosynthetic activity, and (**e**) antioxidant activity.

B has significant involvement in maintaining the stability of the plasma membrane, and B-induced changes in the cell may affect ion transport and other membrane-related processes [76,77,78] (Figure 2a).

In plants with the augmentation of B toxicity under saline conditions, changes in the soluble B concentration in different cell compartments need to be discussed. Few studies have reported high B concentration in the apoplast under B toxicity condition [24], while others studies have reported low B accumulation in the apoplast as compared to that in the symplast [79]. However, this accumulation depends on the plant and its genotypes.

In sunflower, lower B in the apoplasm and higher accumulation of B in the symplasm was observed [79], and it was confirmed that compartmentation did not facilitate the detoxification of surplus B, as boron was not excluded from symplasm but was accumulated in it. This B in the symplasm may lead to the formation of complexes [80] that may develop a concentration gradient between the apoplasm and the symplasm and thus affect the B movement across the membranes.

In wheat, enhanced B accumulation was observed in the apoplasm as compared to that in the symplasm in normal to high B conditions. Consequently, B uptake occurs via passive diffusion through the plasma membrane [77]. In different compartments, B may bind with different molecules to form complexes—for example, it binds with sugar molecules in the cell wall, glycolipids and glycoproteins in the plasma membrane, and RNA, ATP, and NAD molecules in the symplasm [24,81].

Salt supply in the presence of high B increases soluble B concentration in both the apoplasm and the symplasm. This can be attributed to the destruction of cell membrane structure through the production of reactive oxygen species (ROS) and through decrease in the phospholipid content under salinity stress [82,83]. Consequently, it changes the rate of movement of B across the membranes and largely increases the soluble B concentrations in both inter- and intracellular regions. Additionally, soluble B concentration in different compartments is inversely related to the water level of the plants [84]. Hence, salinity stress seems to alter B toxicity symptoms by developing water stress in plants and consequently enhancing the soluble B concentrations in different spaces, thus enhancing the symptoms of B toxicity [26].

Generally, B toxicity tolerance is correlated with decreased B uptake via roots and then to shoots; however, the situation can be different for tolerant genotypes. The tolerant genotypes may show B toxicity tolerance despite higher B uptake and leaf B concentrations. This feature can be attributed to the redistribution of B from the intracellular region to the apoplastic region [85].

In plants with less damaging symptoms of combined BorSal stress, proton transporting ATPases, Na^+^/H^+^ antiporter, and other transporters are thought to be involved. Vacuolar Na^+^/H^+^ antiporter facilitates the movement of Na^+^ ions into the vacuole and may protect the cytoplasm from ion toxicity [86]. Under saline stress condition, vacuolar Na^+^/H^+^ antiporter is overexpressed; this increases the vacuolar compartmentalization of sodium ions and thus increases the salt tolerance in plants [87,88].

The plasma membrane and the vacuolar H^+^-ATPase are the proteins that produce the electrochemical H^+^ gradient across the membranes. This proton gradient facilitates the translocation of sodium ions and Na^+^/H^+^ antiporter out of the cytoplasm and their compartmentation into vacuoles. This in turn may increase the salt stress tolerance by decreasing sodium ion toxicity in the cytoplasm. Several studies have reported that individual salt supply enhances the H^+^-ATPase activity and thus the Na^+^/H^+^ antiport activity in the cell; in contrast, individual boron toxic condition inhibits the H^+^-ATPase activity [21,88,89,90,91]. The combined BorSal condition enhances the H^+^-ATPase activity, although the increment can be lesser than that achieved under the individual salt toxic condition, and it may have an ameliorative effect on plants [21].

Plasma membranes intrinsic proteins (PIPs) including aquaporins control the transport of water into the cells by the formation of aqueous pores in the membranes [92,93,94]. However, in addition to other factors, their expression is also affected by NaCl and B concentrations in the plant cells. Higher NaCl supply increases lipid peroxidation by increasing the membrane permeability and suppressing the intrinsic protein expression [95]; in contrast, increased B supply in combination may reduce the membrane damage due to its role in maintaining the membrane structure, thus balancing the intrinsic protein expression [52,77,96,97] (Figure 2a).

Studies on aquaporin isoforms in maize showed their involvement in the transport of Na and Cl ions and in increasing the membrane permeability towards boric acid. This leads to a reduction in PIP abundance, leading to the regulation of salt and B toxicity. Consequently, PIP aquaporin levels are down-regulated in the presence of salinity with a reduction in PIP abundance. However, these reductions may vary with different isoforms. Likewise, PIP abundance towards B stress also varies in different PIP families. In some studies, transport of boric acid controlled by PIP aquaporins suggests that reduction in PIP levels might diminish the outcomes of boric acid toxicity [21,22,98,99].

## 5. BorSal Interaction in Plants in Terms of Ion Mobility and Exclusion Mechanism

The total Ca^2+^ ion concentrations in plants seem to be a major link in the effect of salinity on B accumulation. Calcium is well known for maintaining the integrity of cell membranes [100]. Salinity stress develops Ca deficiency in plants by disturbing its distribution. It slows down plant Ca^2+^ uptake by disturbing K^+^/Na^+^ balance at membranes [101,102,103]. Salinity either inhibits its movement from root tissues to xylem or hinders its transfer to the leaves [104]. However, increased B concentrations are known to increase Ca^2+^ transport and vice-versa [103,105,106]. As B-based compounds with a negative charge are the most movable forms, positively charged Ca^2+^ ions bind with them and form complexes that migrate faster [24]. Thus, B reduces the diminishing effect of salinity stress on Ca absorption and mobility, thereby strengthening the plasma membranes (Figure 2a).

Imbalanced uptake of inorganic ions such as Na, K, Cl, and B in plants under B and salinity stress has a major role in their tolerance mechanisms [107]. High salinity levels increase Na and Cl absorption, while high B levels decrease their accumulation in plants [60]. High Na import under saline conditions and inhibit the influx of K transporters, thus decreasing the leaf K content of the plants. Hence, this decrease in K content can be attributed to the antagonistic absorption of K with Na in plants [103,104,108]. Several studies have established that, under saline growth conditions, higher K content regulates the normal functioning of the photosynthesis system, and plants with greater leaf K content are more salinity tolerant [23,109,110]. Even after the enhanced uptake of Na, salt-tolerant genotypes may protect themselves against salinity by balancing the intracellular water concentration by compartmentalization, leading to their normal growth [111,112]. B supply does not influence the K^+^ content to a major extent, as it is not involved in the K^+^ uptake pathway [113].

It has been reported, that under high substrate B concentration, the genes encoding root-shoot aquaporins are down-regulated [99]. This decreases the water transport from roots to shoots and consequently decreases the transpiration rate. This in turn regulates the B uptake from soil [99,114]. When B toxicity is accompanied with salinity, the transpiration rate is diminished by both boron toxicity and salt stress and slows down B uptake. Other than the transpiration rate, the exclusion mechanism also affects B uptake from soil. B exporters and boric acid channels at the plasma membrane contribute to export B out of the cell and reduce B toxicity effects [115,116]. Active B efflux from the root cells facilitates low B concentrations in roots and consequently less B accumulation in shoots, leading to high B tolerance in plants [37,117].

Although different characteristics have been discussed related to membrane permeability and its role in providing tolerance to B and salt stress, it also largely varies from species to species and their sensitivity towards salt stress [67].

## 6. BorSal Interaction in Plants in Terms of Water Transport, Transpirational Movement, Photosynthetic Activity, and Changes in Stomatal Resistance

B is mostly immobile in plants, and its movement mainly occurs through xylem depending upon the transpiration force that causes higher B concentration at leaf tips and in mature leaves [8,24]. This leads to a quicker response of B toxicity on older leaves than on younger leaves [45] that spread from the leaf tip towards the base. However, in some plants, B becomes mobile in phloem after forming complexes with polyols, leading to quicker effects on younger leaves and growing tips than on older tissues under B toxic growth conditions [118,119,120].

In a few species, salinity has no influence on B concentrations at the base of the leaves, while it increases the B levels at leaf tips [24]. However, this greatly depends on the plant and the specific genotype. Several genotypes show lower B content in leaves under BorSal stress [50,121]. This occurs with the closing of stomata and decreased transpiration followed by the slow diffusion and the passive absorption of B [53,114] (Figure 2b,d).

Stomatal resistance of leaves is an indicator of stress level in plants [122]. B uptake in plants is a passive process that is largely related to transpiration flow and stomatal conductance [99,114]. High saline conditions lead to water loss in plants that in turn leads to the closure of stomata and induces stomatal resistance, thus hindering the transpiration process. Hence, the excess salt supply under B toxic growth conditions slows down the transpiration process, thereby inhibiting B uptake and translocation [66,97]. Thus, it minimizes the B toxicity symptoms [61,123] (Figure 2b,d).

Moreover, increment in stomatal resistance under saline growth conditions causes a depletion of CO_2_ within the cells that is required for photosynthesis, leading to the accretion of NADPH. Hence, oxygen becomes activated as an electron acceptor forming the superoxide radicals. Although more superoxide radicals can be produced under combined B and salt stress condition, it is followed by the formation of antioxidant molecules under BorSal treatment, leading to the reduction in toxicity symptoms (Figure 2e).

## 7. BorSal Interaction in Plants in Terms of Antioxidant Activity

BorSal stress influences not only the physiological mechanism but also the chemical environment of plants. In B toxicity conditions, ROS production is enhanced [124,125], while under BorSal stress, antioxidants are activated that destroy ROS [97]. Stress conditions lead to the accretion of ROS that causes destruction at the cellular level, resulting in lipid peroxidation of membranes [126]. However, different antioxidants, including ascorbic acid, catalase, and superoxide dismutase, participate in the scavenging of these ROS [127,128,129].

The antioxidant ascorbic acid that scavenges superoxide radicals remains unaltered under the individual B and NaCl treatments; however, its content increases under BorSal treatment [97,130] (Figure 2e). Proline is another indicator of osmotic stress caused by salinity stress in plants. However, its accumulation in plants under B toxic growth conditions is controversial [131]. While some studies report negligible changes in its level under B toxicity, signifying that B toxicity does not lead to osmotic stress [96,97,132], other studies report significant proline accumulation under B toxicity [66,133,134]. In an experiment, Eraslan et al. [97] found that the proline level in lettuce plants did not change under B toxicity; however, an increase in its concentration under salinity and BorSal stress showed that salinity leads to osmotic stress in plants.

## 8. BorSal Interaction in Plants in Terms of pH Changes

pH is one of the factors that might be crucial to understanding salinity–B interactions. B adsorption and its availability to plants are affected by soil pH [52,135]. Chemical speciation of B changes from boric acid to borate ions on changing the soil pH from acidic to alkaline. This alteration influences the membrane transport of B [104,136]. Thus, in saline soil, the reduction process is activated due to increased soil pH, leading to inhibited B uptake (Figure 2c). Accordingly, several studies reported the absence of B toxicity and no yield losses in saline soils [44,137]. Conversely, few studies reported that slightly alkaline conditions are more detrimental for salinity–B interactions and plant growth than slightly acidic conditions. In an experiment conducted on cucumber, increment in B concentration and salinity under slightly alkaline pH of 8 had a mitigating effect on fruit yield. Additionally, similar studies on broccoli showed that high B concentration did not affect the yield at acidic pH 6, while at slightly basic pH of 8, high B concentration caused a significant decrease in yield [18,19,71,73,104]. This could have been due to ineffective chemical speciation of B from boric acid to borate ions under slightly alkaline pH in certain plant species. Moreover, the toxic effects of anionic forms on the plants could also have been a reason for the decrease in yield [18]. Therefore, although making changes in soil pH may work as a supplementary abiotic stress condition and interact differently with other abiotic stresses, it must be considered as a significant variable to understand these interactions [138].

Several studies have been conducted on BorSal interactions where pH conditions were documented [60,107,139,140]; however, few experiments estimated the effect of pH as a variable on this interaction [18].

Although alkaline soil pH is considered to play a role in changing the B form in plants from boric acid to borate, internal slightly basic pH of cellular cytoplasm also controls this conversion. Hence, a less anionic form of B can be obtained in plants, even at high pH [18]. Additionally, other than ionic strength and pH, complex molecules formation of B with phenolic groups regulates its movement across the membranes [141]. Hence, more detailed studies have been suggested at the cellular level to understand the effect of pH on BorSal interactions.

## 9. Molecular Mechanism Involved in B Stress as an Individual Stress Condition

The role of transporters, including channel proteins, carrier proteins, symporters, and antiporters, has been well established in providing tolerance to B and salt toxicity to plants under B and salt stress conditions individually [115,116,142,143].

Pectin found in primary and secondary walls and middle lamella is well known to provide physical strength to plants by joining the cells together [144]. Borate develops ester bonds with two rhamnogalacturonan II (RG-II) molecules that are pectin polysaccharides. Hence, a pectin network of borate-dimerized RG-II molecules is formed that strengthens membrane structure and cell adhesion [145]. Thus, the role of B in the formation of complex networks of pectic polysaccharides is well established at the molecular level [146,147].

B is easily taken up by plants in the form of boric acid and passes through membranes by passive diffusion under normal B growth conditions [148]. Boric acid channels and B transporters play a role in B-deficient and B toxic growth conditions. Although the members of major intrinsic proteins (MIPs) are activated only under deficiency condition, BOR borate exporters are used in both B-deficient and B toxic growth environments [149,150]. Thus, B moves in plants via passive diffusion, boric acid channel-facilitated diffusion, or borate transport [85].

The MIPs of boric acid channels are actively responsible for B transport with its different sub-families involved in different functions. PIPs found in plasma membranes regulate water permeability and dynamics in guard cells of the stomata and the vascular tissues. However, its two isoforms, namely PIP1 and PIP2, act differently, with the former controlling the hydraulic conductivity and the latter participating in cell-to-cell water activity [151]. Nodulin 26-like intrinsic proteins (NIPs) comprise three sub-classes, where the second subclass is considered to act as permease for boric acid and is actively involved in B movement [115,150,152,153,154]. NIPs become more significant under deficient B supply [152].

The *NIP5;1* gene was the first gene to be characterized as boric acid channel after its enhanced expression in *Arabidopsis* under B-deficient conditions [152]. Its GFP-tagged protein GFP-NIP5;1 is confined to the epidermis and the lateral root cap cells under limited B supply [155]. It was also confirmed that NIP5;1-GFP mRNA and protein accumulation is higher in roots under B-deficient conditions; however, the degradation of its mRNA and reduction in its protein accumulation are required for adapting under B toxic condition [156]. *AtNIP6;1*, a paralog of *AtNIP5;1*, is mainly localized in phloem sieve elements, parenchymal cells, and companion cells. It is required for the movement of B from xylem to phloem [156,157]. *NIP7;1* of *Arabidopsis thaliana* that is expressed in pollens of flowers has a lesser role in B transport movement than other boric acid channels. However, conserved tyrosine residue (Tyr81) enlarges the *AtNIP7;1* pore opening that positively affects the B transport activity [158]. Similar to *Arabidopsis* genes, the maize tassel-less1 gene (*ZmTLS1*) is confirmed as a boric acid channel with its involvement in vegetative and inflorescence growth under limited B supply [159]. The *OsNIP3;1* gene of rice is confined to different tissues, including vascular bundles in leaf sheaths and exo-dermal cells in roots. Its function as boric acid channel is confirmed with the reduction in plant growth under B-limiting conditions by the silencing of *OsNIP3;1* RNA [160].

Other than boric acid channels, borate exporters have a crucial role in maintaining the B concentration in cells by the efflux of B. Seven genes have been reported in *A. thaliana* that encode BOR-type borate transporters (BOR1–BOR7) [150,161,162,163] BOR1 is one of the initially determined plasma membrane borate transporters that regulate the B uptake in *A. thaliana* under B-deficient conditions. However, its homologs in yeasts and different plant species confer tolerance towards B toxicity.

BOR1 homologs are known to regulate B uptake by efflux from the root cells to their surroundings and, consequently, their controlled transfer to shoot. In leaves, toxicity symptoms are not always according to the leaf B content because of differential partitioning of B. However, it was suggested that, under toxic conditions, B transporters pump out B from the symplast to the apoplast, where relatively higher B concentrations can be endured. Tolerant cultivars are more efficient in pumping out this toxic B from the cytoplasm to the apoplast [85].

Several studies have reported the role of BOR1 up-regulation in facilitating the movement of B in shoots under B deficient conditions. Recently, Aibara et al. [164] highlighted that controlled post-transcriptional regulation of BOR1 largely allows the plant to avoid B toxicity. The degradation of the BOR1 protein and its translation suppression under B toxic conditions enable the survival of plants by avoiding excess transport of B to shoots. Thus, although BOR4 is accredited with B toxicity tolerance, the regulation of BOR1 is extremely crucial under B-sufficient conditions.

BOR1 homologs in *Saccharomyces cerevisiae* provide tolerance to high B concentration by exporting B out of the cell [117,165,166]. The clones of its orthologs in wheat (*Ta-BOR2*) and barley (*Hv-BOR2*) showed positive associations between their expression levels and the decrease in the B concentrations in roots, specifically in the tolerant cultivars [117,167]. Additionally, they were found to be expressed more in roots than in shoots.

The ortholog of *AtBor1* in wheat, *TaBOR1*, differentially controls the import of B in roots and its movement into shoots [168]. Even its alleles on 5D, 5A, and 5B wheat chromosomes, namely *TaBOR1.1*, *TaBOR1.2*, and *TaBOR1.3*, respectively, express differently under B limiting and B excess conditions. Among these alleles, *TaBOR1.1* and *TaBOR1.3* are upregulated under B deficient conditions; however, *TaBOR1.2* is up-regulated under B toxic conditions in both roots and shoots, signifying that it might be involved in draining out surplus B from the tissues, thus providing endurance against B toxicity.

*AtBOR4* is involved in B exclusion from tissues primarily from roots, leading to excess B tolerance [162,169], and, as compared to AtBOR1, it shares greater amino acid sequence similarity with *TaBor2* and *HvBOR2* [117,167,170]. Previous GUS staining results showed the expression of BOR4 in the root meristems and endodermis of the root hair zone with its polar localization in the outer part of the plasma membrane [162,163]. Being an efflux B transporter, its presence in the outer membrane is likely to alleviate B inflow into xylem.

## 10. Molecular Mechanism Involved in Salinity Stress as an Individual Stress Condition

In most of the plant species, salinity stress can be categorized into two forms, osmotic stress and ion toxicity [112,171,172]. Osmotic stress develops from the decrease in the solute potential in soil with an increase in the salt concentration around the root up to a level of 40 mM NaCl. Consequently, shoot growth is immediately reduced [112]. As NaCl is the most prevalent salt, plants prefer its accumulation as compared to other elements available in low concentrations, such as potassium ions [112]. Moreover, most of the plants are capable of excluding sodium and chloride ions from roots when water is absorbed from the soil [86]. Ion toxicity occurs when over-accumulation of salt (predominantly Na^+^ ions) occurs in the cytoplasm of the plant cells, thereby inhibiting photosynthetic activity, protein synthesis, and other developmental processes [173]. Where on the one hand, osmotic stress is dealt with osmotic tolerance, on the other hand, ionic stress is dealt with Na^+^ exclusion and tissue tolerance [112,173]. Plants can be categorized into two groups, glycophytes and halophytes, where glycophytes are more susceptible to high saline conditions.

The disturbance of Na ions in tissues impedes the K ions in the plants. Hence, salt stress is substantially studied in the context of cytosolic Na^+^/K^+^ ratio [173]. This ratio is largely regulated by the activity of Na and K transporters, especially SOS_1_ (salt overly sensitive 1), HKT (high-affinity K^+^ transporters), and NHX (Na^+^/H^+^ exchangers) transporters. The over- or under-expression of these genes maintain Na ion uptake, their translocation, and homeostasis within tissues [174,175,176,177]. In most of the plant species, SOS_1_, NHX, and HKT transporters are responsible for the efflux of Na^+^ from roots, its sequestration into vacuoles, and its influx in roots along with the recovery from xylem, respectively [178,179,180,181,182,183,184,185]. Although these transporters have their own functions, they collaborate to maintain the processes of the SOS signaling pathway.

The expression of SOS_1_, which encrypts plasma membrane Na^+^/H^+^ antiporter, facilitates xylem loading in roots under limited saline conditions, thereby regulating the distribution of Na ions into shoots and leaves. However, increase in Na+ ion concentration in shoots under high saline environment inhibits their transfer from roots to shoots, thereby minimizing its loading into the root xylem tissues and thus promoting the salinity tolerance of plants [186,187,188,189,190]. It is linked with the efflux of Na^+^ from the root cells to the apoplast with the help of proton gradient developed by H^+^-ATPase [191,192]. This averts the increased concentrations of sodium ions in shoot tissues [182,193]. The reduction of sodium ions accumulation by overexpression of SOS_1_ under high saline conditions has been reported in *Arabidopsis* and other plant species [194,195,196,197].

Other than SOS_1_, two other proteins, SOS_2_ and SOS_3_, are found to be involved in providing salt tolerance to plants [198]. *SOS_2_* gene encrypts a serine/threonine protein kinase and possesses an N-terminal catalytic domain and a C-terminal regulatory domain [199]. SOS_3_ contains a myristoylation location at its N-terminus and a myristoylated Ca+ binding protein, which are both necessary for developing salinity tolerance [200]. Being self-inhibitory in nature, the C-terminal regulatory domain containing FISL motif regulates the kinase activity. The kinase is activated when, under salt stress conditions, the Ca^+^ binding site of SOS_3_ protein binds with the FISL motif of SOS_2_ [201,202,203]. SOS_3_–SOS_2_ complex phosphorylates SOS_1_ and, consequently, activity of the SOS_1_ protein increases, providing salt tolerance to plants [182,204].

HKT transporters promote unloading of Na ions from xylem into parenchymal root cells and loading of K ions from roots into xylem [112,205,206,207,208,209]. Hence, these are some of the chief transporters that regulate Na^+^/K^+^ homeostasis in plants. The up-regulated expression of these genes under high saline conditions occurs with the increase in Na+ concentration in the vacuoles of leaves that causes the discharge of Na from xylem vessels, thus mitigating the salinity effects in leaves [185,210,211]. The two classes of HKT transporters, Class I and Class II, are composed of Na+ specific and Na^+^-K^+^ co-transporters, respectively [212,213]. In a study on *Arabidopsis* plant, Class I transporters HKT1 mutants were found to be susceptible to salinity stress with higher sodium concentration in leaves [185]. Some studies reported that *AtHKT1* intervenes with Na^+^ influx into roots [214], while others informed its role in recovery of Na^+^ from xylem vessels [184]. In rice, *OsHKT2* that belongs to Class II transporters was reported to be involved in both Na^+^ and K^+^ influx into root cells of plants under salinity stress to provide salt tolerance [215,216]. *TmHKT1* of *Triticum monococcum* extracts Na+ from the xylem and regulates movement into leaves [209].

To alleviate Na ion concentration in the cytoplasm, plants sequester Na+ in vacuoles, and NHX (Na^+^/H^+^ exchangers) transporters play a crucial role in this process [182]. The increase in salinity tolerance level through overexpression of NHX transporters has been reported in many plants [217,218,219]. NHX transporters localized in tonoplasts also control the influx of K ions, thereby maintaining the endosomal pH and Na^+^/K^+^ homeostasis [220]. *AtNHX1* and *AtNHX2* of *Arabidopsis* are determined to be involved in K^+^ influx into vacuoles, maintenance of vacuolar pH, and compartmentalization of Na^+^ into vacuoles [220]. The overexpression of NHX transporters under salinity stress and its effect on improving salt tolerance has been widely elucidated in several species, including *Arabidopsis*, wheat, maize, rice, *Brassica*, etc. [218,219,221,222,223,224].

## 11. Common Transporters Involved in BorSal Stress Condition

Different transporters have been studied under individual B and salt stress conditions. Some of these transporters are common under both stress conditions and regulate the toxicity levels by up- or down-regulation of their expression. However, there is a lack of information on their differential expression under a combined stress condition.

Although there have been distinct presumptions on the effect of BorSal stress on plant growth and development, both stress conditions are believed to affect water movement in plants. Thus, aquaporins can be a connecting link in the tolerance mechanism. Aquaporins facilitate the exchange of B through the plasma membrane under high B supply [22] that is largely governed by the alterations in root hydraulic pressure under saline growth conditions. The mitigating effect of salinity on aquaporin activity and thus on B and water influx has been reported through several experiments [10,22,24]. Salt stress causes water deficit, leading to the decrease in the osmotic potential that has a considerable effect on the gene expression of aquaporins or vice-versa [225]. Where different forms of aquaporins are expressed under different stress conditions, plasma membrane aquaporins (PIPs including PIP1; 2, PIP2; 1, and PIP2; 2) largely direct B transport under B-stressed environments [99]. Differential expression of PIPs under BorSal stress directs towards the changes in hydraulic conductivity. However, these changes can be different according to the tolerance levels of cultivars. Moreover, two isoforms, namely PIP1 and PIP2, behave differently under individual and combined B and salt stress conditions. In maize, combined BorSal stress led to a high abundance of *ZmPIP2* and *ZmPIP1* levels in comparison with the individual boric acid and NaCl stress, respectively [21]. In barley, two isoforms of PIPs, namely *HvPIP1* and *HvPIP2*, are recognized as B and salt transporters [226]. A significant reduction in *HvPIP1* and *HvPIP2* activity was found under salt and combined stress in roots as compared to that in B stress alone. However, these isoforms are up-regulated in leaves, thereby maintaining the cellular water level and controlling the water uptake from the soil.

The up-regulation of the SOS_1_ gene in roots and leaves under saline conditions has been reported in different plant species such as alfalfa [227], *Medicago* [228], wheat [229], and maize [230]. However, its expression did not change significantly under BorSal stress. This suggests that SOS_1_ is responsible for removing Na from the roots under saline stress. However, when the salt stress is accompanied by B stress, either other genes mediate the tolerance mechanism or B accumulation in shoots under high B concentration might reduce the movement of B from root to shoot.

Similar to SOS_1_, NHX transporters that move Na+ into vacuoles are not much affected under BorSal stress conditions [231]. However, their activity increases in roots under salt and B stress alone, as they need to separate Na ions in the vacuole to protect the cytosol from toxicity [232]. This could again be due to the regulation of salt toxicity in plants on the application of high B concentration or may be due to the high basal expression level of NHX transporters for the combined stress condition [230].

BOR transporters with the function of expelling B out of root cells and symplast are remarkably down-regulated in maize under combined stress as compared to that under individual salt and B stress. The lesser B content in the shoot of tolerant cultivars with the down-regulation of BOR genes shows that their expression level controls the B uptake and, consequently, its movement into shoots [117,230].

## 12. Conclusions

From different studies, it can be understood that a combined B and salinity stress condition does not always have a positive effect on plant development as compared to individual stresses. Hence, the involved genes should be studied at the molecular and the cellular levels by comparing the mechanism under two conditions—one where combined stress has a positive effect on plants and another where combined stress has a negative effect on plants. Moreover, most of the gene expression-based BorSal stress studies have been performed on tolerant cultivars. The comparison of gene regulation in tolerant and susceptible cultivars may highlight new pathways in B and salt transport in plants. Additionally, the same plant species demonstrated different outcomes under BorSal stress in different experiments. Although the growth conditions involving soil, temperature, B, and salt dosages were different in these experiments, the genetic variation of cultivars cannot be neglected. Hence, it is suggested to compare the gene expression results of different cultivars developed under the same high B and salt stressed growth environment. The genetic variation of these cultivars may give new insights into the responsible pathways in terms of expression of the involved genes. The differential regulation of a gene can be responsible for the susceptibility of a cultivar to a particular stress condition. A hypothesis can be considered that, if a gene is up-regulated or vice-versa under BorSal stress, stimuli that can oppositely express that gene should be investigated. For example, if any gene A is down-regulated in a susceptible cultivar under BorSal stress, the factors that can up-regulate its expression in the susceptible cultivar should be discovered. Furthermore, it should also be investigated whether the upregulation of that gene A in susceptible cultivar improves its tolerance to BorSal stress. The purpose of this review was to emphasize that BorSal stress should be considered as a novel stress condition, and the information provided in this review can be used to understand the gaps in this research area. These gaps should be closed so that crops can be grown under combined B and salt-stressed agricultural fields and yields can be elevated.
